# Isolation and identification of *Asaia* sp. in *Anopheles* spp. mosquitoes collected from Iranian malaria settings: steps toward applying paratransgenic tools against malaria

**DOI:** 10.1186/s13071-018-2955-9

**Published:** 2018-06-28

**Authors:** Abbas Rami, Abbasali Raz, Sedigheh Zakeri, Navid Dinparast Djadid

**Affiliations:** Malaria and Vector Research Group (MVRG), Biotechnology Research Center (BRC), Pasteur Institute of Iran, Tehran, Iran

**Keywords:** Paratransgenesis, *Asaia*, Bacteria, Malaria, Vector-borne diseases, Vector control

## Abstract

**Background:**

In recent years, the genus *Asaia* (Rhodospirillales: *Acetobacteraceae*) has been isolated from different *Anopheles* species and presented as a promising tool to combat malaria. This bacterium has unique features such as presence in different organs of mosquitoes (midgut, salivary glands and reproductive organs) of female and male mosquitoes and vertical and horizontal transmission. These specifications lead to the possibility of introducing *Asaia* as a robust candidate for malaria vector control *via* paratransgenesis technology. Several studies have been performed on the microbiota of *Anopheles* mosquitoes (Diptera: Culicidae) in Iran and the Middle East to find a suitable candidate for controlling the malaria based on paratransgenesis approaches. The present study is the first report of isolation, biochemical and molecular characterization of the genus *Asaia* within five different *Anopheles* species which originated from different zoogeographical zones in the south, east, and north of Iran.

**Methods:**

Mosquitoes originated from field-collected and laboratory-reared colonies of five *Anopheles* spp. Adult mosquitoes were anesthetized; their midguts were isolated by dissection, followed by grinding the midgut contents which were then cultured in enrichment broth media and later in CaCO_3_ agar plates separately. Morphological, biochemical and physiological characterization were carried out after the appearance of colonies. For molecular confirmation, selected colonies were cultured, their DNAs were extracted and PCR was performed on the *16S* ribosomal RNA gene using specific newly designed primers.

**Results:**

Morphological, biochemical, physiological and molecular results indicated that all isolates are members of the genus *Asaia.*

**Conclusions:**

Contrary to previous opinions, our findings show that *Asaia* bacteria are present in both insectary-reared colonies and field-collected mosquitoes and can be isolated by simple and specific methods. Furthermore, with respect to the fact that we isolated *Asaia* within the different *Anopheles* specimens from distinct climatic and zoogeographical regions, it is promising and may be concluded that species of this genus can tolerate the complicated environmental conditions of the vector-borne diseases endemic regions. Therefore, it can be considered as a promising target in paratransgenesis and vector control programs*.* However, we suggest that introducing the new technologies such as next generation sequencing and robust *in silico* approaches may pave the way to find a unique biomarker for rapid and reliable differentiation of the *Asaia* species.

## Background

Malaria is considered as the most deadly parasitic communicable disease worldwide and the most important vector-borne disease in Iran. Furthermore, it is a major health challenge in northern and southern parts of Iran [1, 2]. The WHO world malaria report in 2015 proclaimed 212 million cases of disease and 429,000 deaths worldwide in 2015 [3]. Widespread insecticide and drug resistance in mosquitos and parasites, respectively, and the lack of an effective malaria vaccine are the main obstacles to achieve the goals of global malaria eradication program [4, 5]. It is obvious that new solutions and management strategies are required for a successful control of malaria disease [6]. To overcome the existing barriers, the WHO invited about 2500 scientists with distinct specialties in different scientific groups from all over the world to determine the drawbacks of the first global malaria eradication program and suggest their solutions to revise the existing program and achieve global malaria eradication by 2050 [6]. One of the scientific groups was related to the vector control. They believed that to support the efforts towards global malaria elimination and eradication, development of complementary vector control strategies are mandatory. Particularly, those that are less vulnerable to the current problem in vector control and insecticide resistance are mandatory and these strategies should be considered in the frontline of malaria vector control intervention approaches. Therefore, these scientists suggested that the main attempts in vector control should be focused on transgenesis and paratransgenesis approaches, and designing new and efficient insecticides with different effect mechanisms compared to previous ones [7].

The main goal of performing the vector control approaches is intervening the transmission of parasites through the mosquitoes [8]. This aim can be achieved by killing the mosquitoes with insecticides and affecting the fitness of mosquitoes or reducing the competency of the vector by presenting new effector molecules which consequently block the sexual parasite development. In recent years, transgenesis and paratransgenesis have been introduced as promising strategies which can be used as effective tools for controlling vector-borne diseases. Genetically modified mosquitoes that express anti-plasmodium effector molecules in their midguts have been introduced in recent years [9–12]. Although genetic modification of insects was a challenge in previous years, new approaches such as phi C31 and CRISPR/Cas9 have been recently introduced and have improved the efficacy of the process [13–16]. Nevertheless, creation of genetically modified insects needs advanced technology, special instruments and skills. In addition, this technology reduces the risks and concerns of gene flow from one mosquito population to another which can be possible in the future [8, 17]. Another interesting technique for delivering the effector molecules is the genetic engineering of symbiotic microorganisms of mosquitoes such as bacteria, fungi and viruses to present the interfering molecules which is also known as paratransgenesis [5, 18–24]. Several studies have been performed on identification and using competent microorganisms to combat against vector-borne diseases, especially against malaria [1, 2, 25–29].

Among the mentioned microorganisms, bacteria have specific advantages such as: simple isolation, growth in basic media, availability of genetic engineering methods and tools, rapid replication rate and relatively uncomplicated approaches compared to other candidate microorganisms. Therefore, bacteria have been on the frontline of attention for developing an effective paratransgenic tool against malaria from the creation of this concept. Engineered vector-associated bacteria could impose a pathogenic effect on their host, interfere with their reproduction and fitness or reduce their competency [8, 30]. *Asaia* is a genus of bacteria with unique features which has been isolated and characterized from the malaria vectors. This bacterium was isolated from *An. stephensi* for the first time and genetically modified and labeled by green fluorescent protein by Favia et al. [7]. Consequently, it was revealed that the bacteria are localized in many organs such as the midgut, salivary glands, and the reproductive system of female and male *Anopheles* and can disperse between these organs through the hemolymph [8]. Furthermore, Favia et al. [8] demonstrated that *Asaia* can transmit to the next generation vertically. These special features are the main advantages of this bacterium and those have led to *Asaia* be considered as a versatile tool in paratransgenesis. According to the performed studies, the distribution of these bacteria in mosquito populations is performed through various mechanisms, including co-feeding, sexual mating and maternal transmission [31, 32]. *Asaia* can be cultivated and genetically modified in the laboratory and can also be recolonized in the new host [33]. Vertical transmission capability suggests that *Asaia* is able to establish stable associations across multiple generations. This bacterium can be cultivated in cell-free media and can be easily transformed with exogenous DNA [8]. In a previous study, we could not isolate *Asaia* from field-collected samples from the north and south regions of Iran. According to the aforementioned specifications of *Asaia* and its importance in paratransgenesis, we modified our previous methods and tried to isolate *Asaia* in normal flora of different species of *Anopheles* in different regions of Iran, including Mazandaran, Bandar-Abbas, Chabahar and Kazerun*.*

## Methods

### Field collection of *Anopheles* spp.

Mosquitoes which were used in this study, originated from field- and laboratory-reared colonies of adult *Anopheles* spp. Coastal, semi-mountainous, or hilly rural areas (Siaho area and Eslamabad) with many permanent and seasonal rivers are the most important urban and rural regions for catching *Anopheles* in north, south, and southeastern part of Iran. Therefore, these regions with a wide distribution were selected for sampling and specimens were collected from Mazandaran, Chabahar, Bandar-Abbas (Siaho area) and Kazerun (Eslamabad) districts (Table 1). Larvae were collected from larval habitats using the standard dipping technique (350 ml dipper) and adults were captured from human and animal refuges and human settlements by hand catch method using an aspirator. After sampling, the larva specimens were transferred alive to the National Insectarium of Malaria and Vector Research Group (MVRG) at the Pasteur Institute of Iran (Tehran). After maturation, their adults were identified to species level using the standard morphological key of Iranian anophelines to determine the species of all *Anopheles* samples [34].

**Table 1:** *Anopheles* spp. studied, their collection sites, number of the mosquitoes and the prevalence of *Asaia* in their midguts

Species	Bandar-Abbas	Chabahar	Mazandaran	*Kazerun*
*An. stephensi*	38/50 (70%)	43/50 (86%)	0	0
*An. maculipennis*	0	0	7/10 (70%)	0
*An. superpictus*	0	0	0	9/14 (64%)
*An. fluviatilis*	0	0	0	7/16 (43%)
*An. dthali*	8/11 (73%)	0	0	0

### Dissection of mosquitoes, isolation and cultivation of microorganisms

**Fig. 1:**
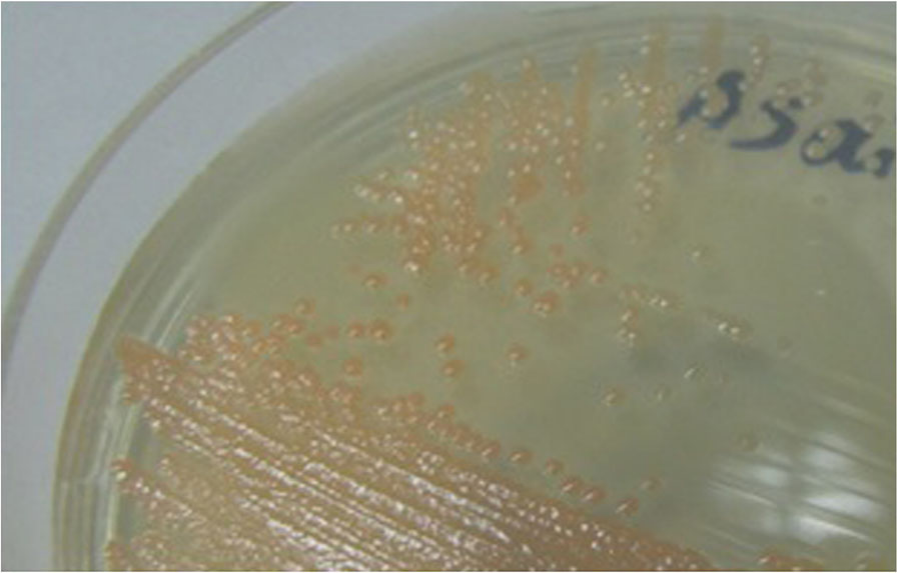
The appearance of *Asaia* colonies on the glucose-yeast extract-CaCO_3_ medium. *Asaia* species produce the pink to colorless colonies on glucose-yeast extract-CaCO_3_ medium which is one of their unique morphological characteristics

All adult mosquitoes were anesthetized by incubating them for 2 min at -20 °C. Before dissection, the surfaces of the samples were sterilized by soaking in 70% ethanol in 2 ml micro-tubes and shaking for 5 min. Dissection was accomplished in sterile conditions under a biological laminar flow hood (class-II). Each dissected midgut was transferred to a 1.5 ml micro-tube; its contents were suspended in 1 ml of sterile saline solution (0.9% NaCl) and homogenized by an electrical homogenizer. Then, a 0.5 ml aliquot of this suspension was inoculated into the handmade enrichment media [8]. A specific enrichment broth media was used for isolation of the acetic acid bacteria in this study comprising 2.2% D-sorbitol, 0.5% peptone, 0.5% yeast extract (Merck, Darmstadt, Germany), 100 ppm cycloheximide and its pH was adjusted to 3.5 with hydrochloric acid [8, 33, 35–37]. Next, isolated bacteria were inoculated in 10.0 ml of handmade enrichment culture medium in culture tubes. When microbial growth occurred, the extent of the growth was determined by turbidity monitoring. After that the suspension of the microorganisms was rubbed on a CaCO_3_ agar plate which contained 2.2% D-glucose, 1.0% ethanol, 1.0% yeast extract, 0.7% CaCO_3_ and 1.2% agar [36, 37]. *Asaia* colonies were experimentally identified by the morphological properties and formation of carbonate dissolution haloes in agar plates (Fig. 1). Colonies which were capable of clearing the CaCO_3_ were selected and isolated to perform more experiments and characterization. The isolates were maintained on agar slants of AG medium which contained 0.1% D-glucose, 1.5% glycerol, 0.5% peptone, 0.5% yeast extract, 0.2% malt extract, 0.7% CaCO_3_ and 1.5% agar [36].

### Morphological, biochemical and physiological characterization

Gram stain was used to study the morphological characteristics of bacteria. The presence of catalase was tested by adding a few drops of 3.0% hydrogen peroxide solution on the bacterial colonies. Assimilation of ammoniacal nitrogen was examined by testing the growth of isolates at 30 °C for 5 days on a medium which contained 3.0% D-glucose, 0.1% (NH_4_)_2_SO_4_, 0.01% K_2_HPO_4_, 0.09% KH_2_PO_4_, 0.025% MgSO_4_.7H_2_O, 0.0005% FeCl_3_.6H_2_O and its pH was adjusted to 4.0 and 7.0 separately [36]. In addition, growth ability was tested in AG-medium without CaCO_3_ and agar as basal medium which contained various concentrations of acetic acid up to 0.35% at 30 °C for five days. Acid production from sugars was tested by the method of Asai et al. [37].

### DNA extraction from bacterial isolates and amplification of *16S* rRNA gene

The DNA of bacterial colonies was extracted with a Cinna Pure DNA kit according to the manufacturer’s instruction (Cinna Gen, Tehran, Iran). Specific forward and reverse primers were designed based on the specific *16S* rRNA gene fragments of *Asaia* bacteria using the Gene Runner software (version 5.0.59) as follows: 16sF (5'-TGG CGG ACG GGT GAG TAT C-3'), 16sR (5'-AGT TGG TTT GAC CCG AAG CC-3') based on the LT838398.1 GenBank accession number and Asafor (5'-GCG CGT AGG CGG TTT ACA C-3') and Asarev (5'-AGC GTC AGT AAT GAG CCA GGT T-3') were used based on our previous study [2]. 16sF-16sR and Asafor-Asarev primer pairs were used to amplify the 1370 bp and 180 bp fragments, respectively. Reaction mixtures were prepared with a final volume of 25 μl using 1 unit of Dream Taq DNA polymerase (Fermentas, Waltham, USA), 0.25 mM dNTPs, 1× Taq polymerase buffer, 0.4 μm of each primer, and 20 ng of the extracted DNA as template. Reactions were performed at 94 °C for 5 min and cycled 35 times through a protocol of 30 s at 94 °C, 30 s at 60 °C, and 100 s at 72 °C. Finally, the reactions were maintained at 72 °C for 10 min. Amplicon size was confirmed by agarose gel electrophoresis on 1% agarose gel. Staining was done with ethidium bromide and amplicons were visualized by an UV trans-illuminator.

### Cloning

Amplified fragments were purified by the Accu Prep® Gel Purification Kit according to the manufacturer’s instruction (Bioneer Corporation, Seoul, Korea) and PCR products with the expected size were cloned into the pDrive TA cloning vector (Qiagen, Hilden, Germany).

### Sequencing

The TA-cloned inserted sequences (six samples) were sequenced by the Millegene Company (Labege, France). Sequences were further analyzed for confirmation using the nucleotide BLAST (http://blast.ncbi.nlm.nih.gov/Blast.cgi) and those were submitted to GenBank (Table 2).

**Table 2:** Information about the isolated bacteria: colony codes of the isolated *Asaia* bacteria, *Anopheles* spp. from which bacteria were isolated, their collection sites and the accession numbers of the *16S* rRNA gene sequence of the isolated *Asaia* species

Colony code	*Anopheles* spp.	Collection site	GenBank ID
Colony13	*An .stephensi*	Bandar-Abbas	KU529464
Colony31	*An .stephensi*	Chabahar	KU529465
Colony33	*An. maculipennis*	Mazandaran	KU529466
colony41	*An. superpictus*	Kazerun	KU529467
colony57	*An. fluviatilis*	*Kazerun*	KU529468
colony95	*An. dthali*	Bandar-Abbas	KU529469

### Phylogenetic analysis

In the next step, these six sequences were aligned using the MEGA6.0 software and Clustal X program with the available sequences representative of *Asaia krungthepensis*, *Asaia bogorensis*, *Asaia platycodi*, *Asaia siamensis*, *Asaia lannaensis*, *Asaia prunellae*, *Asaia spathodeae* and *Asaia astilbes* for genetic linkage analysis. Additionally, three species of the family *Acetobacteraceae* including *Neoasaia chiangmaiensis*, *Gluconacetobacter takamatsuzukensis* and *Acetobacter aceti* were selected as outgroup taxa. Phylogenetic trees were constructed using the neighbour-joining (NJ) method. The NJ analysis was performed using the Tamura-Nei model with MEGA6.0 software [38]. The statistical robustness of the clusters was evaluated by bootstrap analysis after 1000 replications.

## Results

As indicated in Table 1, *Asaia* bacterium was isolated with various prevalence from the field-collected *Anopheles* species from different regions of Iran. All the isolates were gram-negative, coccobacilli-shaped and strictly aerobic (require a shaking incubator) and their size ranges were 0.4–1.0 × 0.8–2.0 μm. Isolates had swarming when those were cultured and streaked on the agar medium. Colonies were pink to yellowish and white, smooth, glossy which had been marked by a full margin on glucose-yeast extract-CaCO_3_ agar plates (Fig. 1). Good growth occurred at pH 3.0 (titration with hydrochloric acid instead of acetic acid) and 30 °C. All new isolates grew on mannitol, dulcitol, D-sorbitol, glycerol, and maltose agar, but none of them grew in the presence of ethanol (Table 3). In addition, the isolates did not produce a water-soluble dark pigment on the glucose-yeast extract-CaCO_3_ medium. Acetic acid allowed a little growth at 0.3% but at 0.35%, growth was inhibited completely. Adequate growth was achieved by the use of D-glucose and ammonium sulfate as the sole sources of carbon and nitrogen on the vitamin-free medium.

**Table 3:** Distinctive features of the genus *Asaia* and other acetic acid bacteria genera [35, 42] applied for identification of *Asaia* within *Anopheles* species

Feature	*Asaia*	*Acetobacter*	*Gluconobacter*
Assimilation of ammonium sulfateon glucose medium	+	+w	-
Acetic acid production on ethanol-CaCO_3_ agar	- or +w	+	+
Acid production from			
D-mannitol	- or +	- or +w	+
D-sorbitol	- or +	-	+
Dulcitol	+	-	-
Glycerol	+	-	+
Ethanol	- or +w	+	+
Maltose	+	-	-

*Key*: w, weak reaction; +, ability to use substrate; -, inability to use substrate

**Fig. 2:**
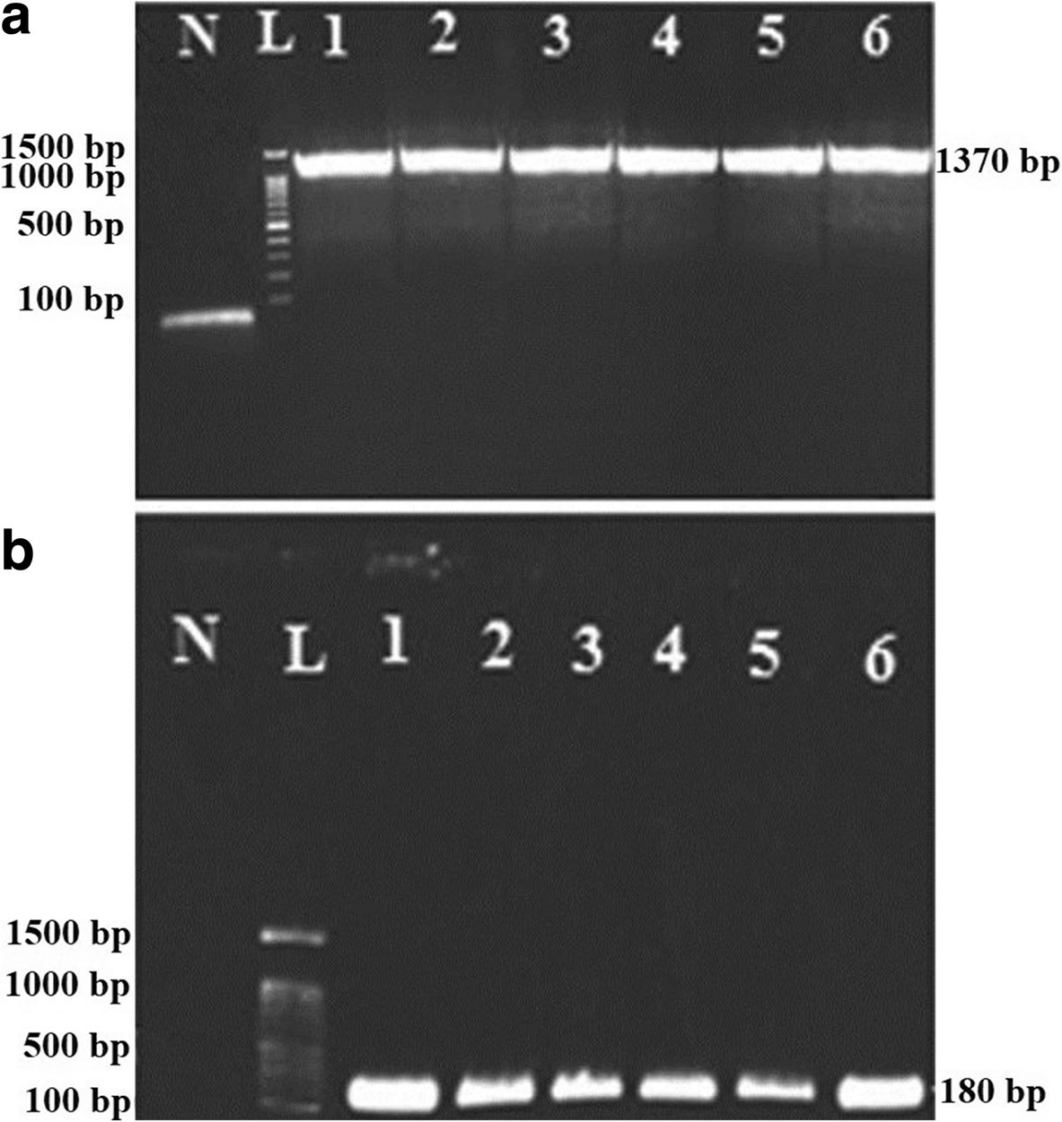
Agarose gel electrophoresis of the *16S* rRNA gene amplicons of *Asaia* sp. Electrophoresis of the 1370 bp (**a**) and 180 bp (**b**) amplicons which correspond to the general and specific amplicons of *16S* rRNA in the genus *Asaia*. Lane N: negative control; Lane L: DNA marker (100–1500 bp); Lane 1: *Asaia* bacterium from *An .stephensi* (Bandar-Abbas); Lane 2: *Asaia* bacterium from *An .stephensi* (Chabahar); Lane 3: *Asaia* bacterium from *An. maculipennis* (Mazandaran); Lane 4: *Asaia* bacterium from *An. superpictus* (Kazerun); Lane 5: *Asaia* bacterium from *An. fluviatilis* (Kazerun); Lane 6: *Asaia* bacterium from *An. dthali* (Bandar-Abbas)

As mentioned above, for molecular characterization and confirmation, we designed two sets of *Asaia 16S* rRNA gene-specific primers. After DNA extraction of the isolated strains, PCR was performed with two sets of primers separately. Following the agarose gel electrophoresis, the 1370 bp and 180 bp specific amplicons of *Asaia 16S* rRNA gene were observed (Fig. 2). PCR analysis confirmed the presence of *Asaia* spp. in nearly 70% of the different *Anopheles* specimens from distinct regions of Iran (Table 3).

These amplicons were recovered from agarose gel, TA-cloned and sequenced. Analysis of these sequences with nucleotide BLAST revealed that they were related to the *16S* rRNA gene of *Asaia*.

Among these sequences, six were submitted to the GenBank database with the accession numbers KU529464-KU529469. The details of each sequence such as geographical sources and species of the *Anopheles* are presented in Table 2.

**Fig. 3:**
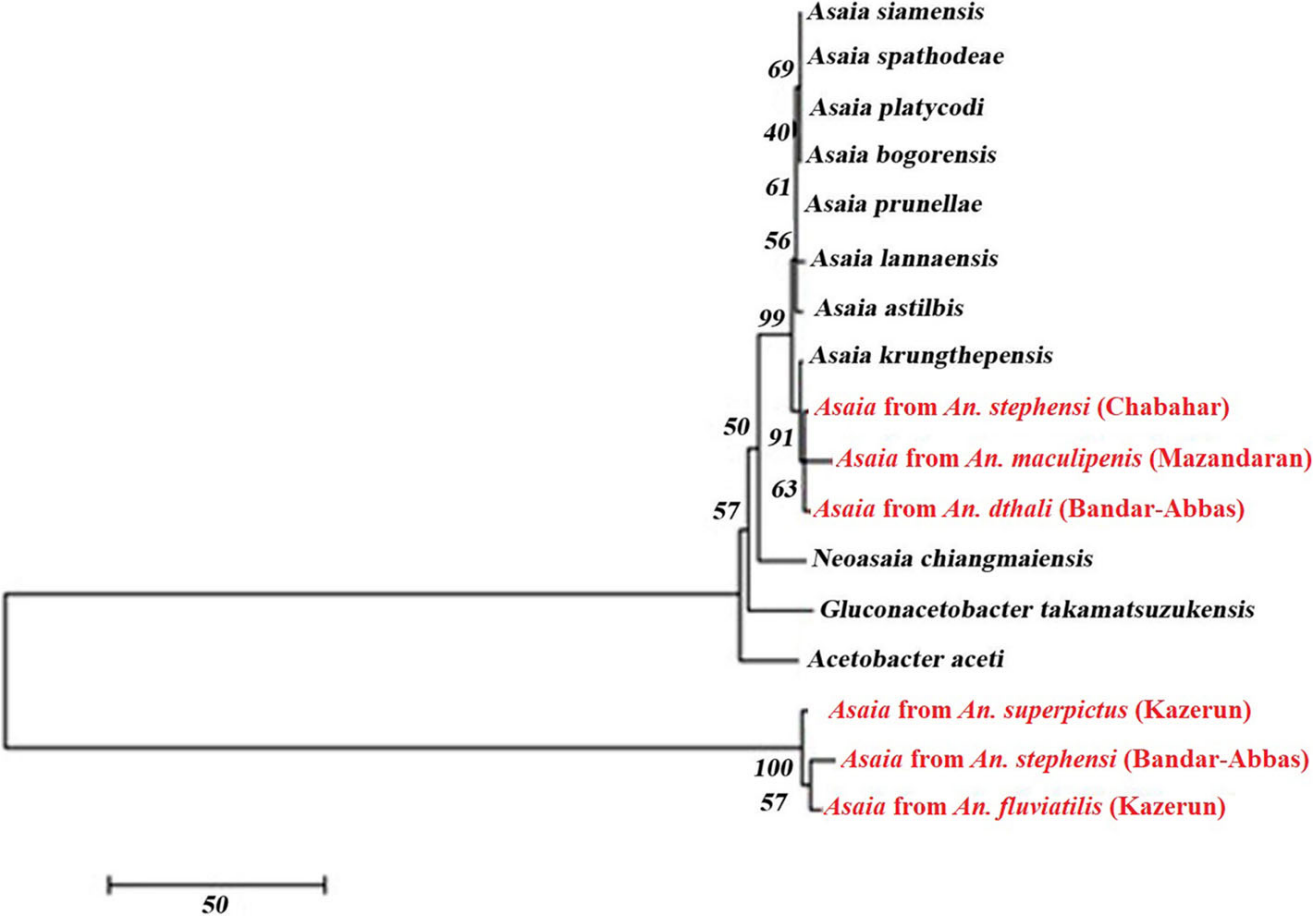
Evolutionary analysis of the isolated bacteria. Phylogenetic tree created with MEGA6.0 software and ClustalX program based on *16S* rRNA gene sequences. The neighbor-joining method was used for evolution relationship analysis and associated taxa were clustered together in the bootstrap test (1000 replicates)

In the next step, these six sequences were aligned using the MEGA6.0 software and Clustal X program with the available sequences of different species of *Asaia* in the GenBank database. The resulting dataset of 1300 nucleotides was used to infer the genetic relationships of the under study samples using a phylogenetic approach based on the NJ method. The constructed phylogenetic tree showed that our isolated samples were not completely similar and located in different clusters. Among them, *Asaia* isolates from *An. stephensi* (Chabahar), *An. maculipenis* (Mazandaran) and *An. dthali* (Bandar-Abbas) clustered with known *Asaia* spp. and revealed a close relationship with *Asaia krungthepensis*. Moreover, it was interesting that *Asaia* isolates from *An. superpictus* (Kazerun), *An. stephensi* (Bandar-Abbas) and *An. fluviatilis* (Kazerun) clustered separately from all the other samples (Fig. 3).

## Discussion

Paratransgenesis is a novel approach which has been introduced by the scientific community as an effective and promising method for controlling the vector-borne diseases such as malaria in recent years [39–41]. Among the several candidates which have been presented for combating malaria, *Asaia* is at the focus of considerations due to its specific features such as horizontal and vertical transmission and moving through the hemolymph inside the mosquito’s body towards different organs. In the present study, *Asaia* sp. was isolated from the midgut of different *Anopheles* mosquitoes using the selected medium to provide the basic element for developing a robust tool against malaria.

A variety of pink-colored to colorless colonies which are observed in frequent culturing are the probable marker of growth for this bacterium [36]. *Asaia* is the only genus of the family *Acetobacteraceae* that assimilates ammonium sulfate on glucose medium. Species of this genus are not fast-growing on ethanol-CaCO_3_ agar, and unlike species of the other genera, do not use ethanol [42]. However, acid production from dulcitol and maltose is helpful for differentiation between the *Asaia* and other genera (Table 1). In addition to morphological and biochemical characterization of *Asaia* in this study, the isolated strains were confirmed with specific primers for the *16S* rRNA gene.

Sequence analysis of the *16S* ribosomal gene confirmed that all of these strains belonged to the genus of *Asaia*, but because the *16S* rRNA gene had high similarity among different species of this genus, the current molecular method could not differentiate these species. Regarding the fact that there is no record on preference of using a specific species of *Asaia* for paratransgenesis, it is necessary to evaluate their efficacy and compare the level of unique features of each species within this genus to select the best one for future paratransgenesis studies. Therefore, the biochemical tests carried out in the present study can be used to differentiate some species of the genus *Asaia*. For instance, unlike other species that do not grow on maltose, only this bacterium has the ability to grow in a maltose-containing environment [43]. Furthermore, the species *Asaia bogorensis* and *Asaia siamensis* which are not able to produce acetic acid from ethanol on the ethanol/calcium carbonate agar, instead can produce acid from dulcitol, glycerol and maltose [36, 43–45].

Sequence analysis of the *16S* ribosomal RNA gene of isolated strains in this study, confirmed the similarity and relationship of some of *Asaia* isolates which were detected in *Anopheles* from Mazandaran (*An. maculipenis*), Chabahar (*An. stephensi*), and Bandar-Abbas (*An. dthali*) with *A. krungthepensis* (Fig. 3). Furthermore, other *Asaia* samples which were isolated from *Anopheles* from Kazerun (*An. superpictus* and *An. fluviatilis*) and Bandar-Abbas (*An .stephensi*) clustered separately from the other isolated specimens in the phylogenetic tree (Fig. 3).

To the best of our knowledge, this is the first study of simultaneous isolation, identification and characterization of *Asaia* within a variety of *Anopheles* species, including *An. maculipenis*, *An. stephensi*, *An. dthali*, *An*. *superpictus* and *An. fluviatilis* from different zoogeographical and vector-borne disease-endemic areas in the world.

One of the major achievements of this study was that we could isolate *Asaia* from the insectary-reared colonies (*An .stephensi* from Chabahar and Bandar-Abbas). This achievement shows that *Asaia* can tolerate different situations and confirmed its vertical and horizontal transmission through several generations. With regard to the importance of *Asaia* in paratransgenesis, its isolation, identification, and characterization from various species of *Anopheles* would be beneficial and could be an applied step toward achieving the applying of paratransgenesis against malaria. Several studies have been performed for isolation of *Asaia* from different medically important vectors and this bacterium has been isolated from *An. gambiae*, *An. stephensi*, *Aedes aegypti*, *Aedes albopictus*, *Culex pipiens* and *Culex quinquefasciatus* [46–48]. This study is, however, the first report of isolation of *Asaia* from *An. fluviatilis*, *An. maculipenis* and *An. dthali* and the first report of its isolation from *An. stephensi* in Iran. However, to determine which species is dominant in each mosquito species and which of them are more efficient for using in paratransgenesis, it seems that developing new molecular or biochemical approaches are necessary.

## Conclusions

In this study, samples for species of the genus *Asaia* were successfully isolated and identified from different *Anopheles* mosquito species with various vectorial capacities and from different vector-borne disease endemic regions. Phylogenetic analysis revealed that the position of the isolated *Asaia* samples is close to other known species of this genus. However, with the present data, determining the species of *Asaia* is not possible and there is a necessity for developing a novel, reliable method to differentiate various species within this genus. Introducing new technologies such as next generation sequencing and robust *in silico* approaches, are the promising points that may make it possible to find a unique biomarker for rapid and reliable differentiation of the *Asaia* species.
